# An insight into novel therapeutic potentials of taxifolin

**DOI:** 10.3389/fphar.2023.1173855

**Published:** 2023-05-12

**Authors:** Yang Liu, Xiaolu Shi, Ye Tian, Shaobo Zhai, Yuyan Liu, Zhengrong Xiong, Shunli Chu

**Affiliations:** ^1^ Department of Implantology, Hospital of Stomatology, Jilin University, Changchun, China; ^2^ Department of Endodontics, Hospital of Stomatology, Jilin University, Changchun, China; ^3^ Polymer Composites Engineering Laboratory, Changchun Institute of Applied Chemistry, Chinese Academy of Sciences (CAS), Changchun, China

**Keywords:** taxifolin, chemotherapeutics, antioxidant, anti-inflammation, flavonoids

## Abstract

Taxifolin is a flavonoid compound, originally isolated from the bark of Douglas fir trees, which is often found in foods such as onions and olive oil, and is also used in commercial preparations, and has attracted the interest of nutritionists and medicinal chemists due to its broad range of health-promoting effects. It is a powerful antioxidant with excellent antioxidant, anti-inflammatory, anti-microbial and other pharmacological activities. This review focuses on the breakthroughs in taxifolin for the treatment of diseases from 2019 to 2022 according to various systems of the human body, such as the nervous system, immune system, and digestive system, and on the basis of this review, we summarize the problems of current research and try to suggest solutions and future research directions.

## 1 Introduction

Medicinal plants have received a lot of attention recently for their widely application in inhibiting and/or mitigating cancers, inflammation, cardiovascular diseases and neurodegenerative diseases. Besides, they have demonstrated remarkable potential in controlling Corona Virus Disease 2019(COVID-19) (Heim et al., 2002; Mahomoodally et al., 2005; Stenger Moura et al., 2019).

Flavonoids, which have been widely recognized as active components of many medicinal plants, can protect the plants from biotic and abiotic stresses, and are associated with the prevention of certain degenerative diseases (Pew, 1948). The variety of pharmacological activities and therapeutic potential of flavonoids are attributed to their degree of hydroxylation, structural class, other substitutions and conjugations, degree of polymerization, and also metal chelation activities (Heim et al., 2002). Several pharmacological activities are associated with flavonoids, including antioxidant (Cao et al., 1997), anti-inflammatory (Elliott and Chithan, 1994), anticancer (Elliott and Chithan, 1994), and cardioprotective properties (Middleton et al., 2000), *etc.*

The flavonoid taxifolin, also known as dihydroquercetin, has similar pharmacological effects to other flavonoids. Its antioxidant capacity, however, is superior to that of common flavonoids (Zu et al., 2014), and it is widely distributed and abundant, which is why its medicinal value is gradually becoming recognized (Chen et al., 2017). In practice, It is rarely used singly, but is often used in preparations such as silymarin (Legalon™) along with silybin A, silybin B, isosilybin A, etc (Klein et al., 1998; Ding et al., 2001). With the exploration of its resources and medicinal potential goes deeper, the therapeutic efficacy and the medical value of taxifolin is becoming unambiguous. To help the future research, this article, after a review on pharmacological activities, focuses on the breakthroughs on the applications of taxifolin in treatment of diseases across human systems from 2019 to 2022, and look ahead the future therapeutic potentials of taxifolin.

## 2 Taxifolin and its pharmacological activities

**FIGURE 1:**
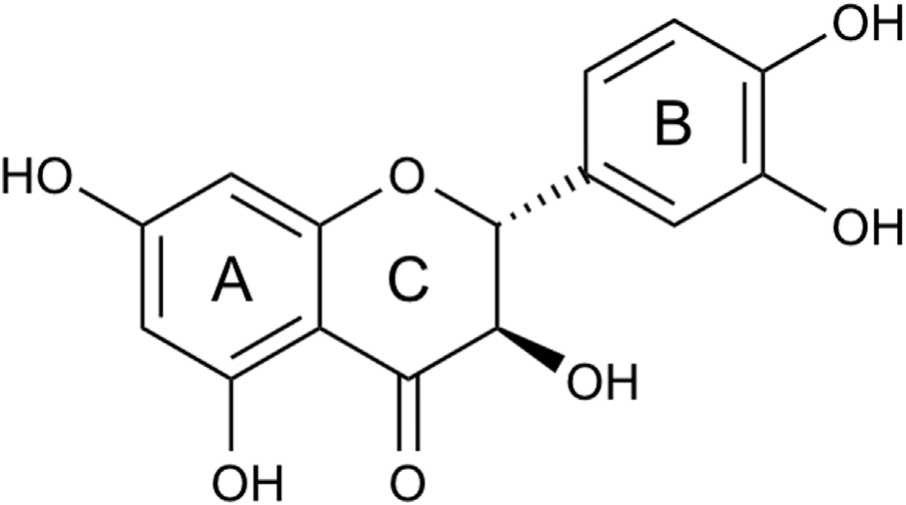
Structure of taxifolin. Taxifolin consists of two phenyl groups **(A, B)** ring, which are joined by a heterocyclic ring **(C)** ring.

Originally isolated from the bark of Douglas fir trees, taxifolin also exists in a variety of plants, such as larch, camphor pine, water safflower, and olive oil (Liu et al., 2014). The molecular weight of taxifolin is 304.25, and the molecular formula is $C_{15}H_{12}O_{7}$ (PubChem, 2023). Shown in Figure 1, taxifolin consists of two phenyl groups A and B ring, which are joined by a heterocyclic ring (C ring) (Ibrahim, 2000; Beecher, 2003) (Figure 1). The complex structure gives taxifolin a variety of pharmacological activities, the most fundamental among which are antioxidant and anti-inflammatory properties.

### 2.1 Antioxidant activity

The structural diversity of flavonoids is thought to be an influential factor in their antioxidant activity (Zeng et al., 2020). There is a direct link between the number of hydroxyl groups attached to the aromatic ring and the antioxidant activity of the molecule (Galato et al., 2001). Taxifolin is a naturally bioactive flavonoid with superior antioxidant capacity than common flavonoids, which is closely related to its phenolic hydroxyl groups (Sroka and Cisowski, 2003; Zu et al., 2014). The 5- and 7-OH groups present in the A- and C-rings have a 4- oxo function, which allows taxifolin to exhibit a powerful scavenging effect on free radicals (Salah et al., 1995). In addition, the strong oxidative properties of taxifolin also result from its conjugated structure and the resonance stability of the two phenolic rings (Topal et al., 2016). However, due to the absence of C2, C3-double bond in the C ring of taxifolin, its antioxidant activity is still slightly inferior to that of other flavonoids with identical hydroxylation pattern lacks antioxidant potency (Sunil and Xu, 2019).

Taxifolin is an effective ⋅OH scavenger that may protect bone marrow-derived mesenchymal stem cells (bmMSCs) from injury caused by ⋅OH. Further using various antioxidant assays to identify possible mechanisms, taxifolin was observed to be effective in scavenging ⋅ OH, DPPH ⋅ and 〖ABTS〗^+ ⋅ radicals and increasing relative 〖Cu〗^(2+)- and 〖Fe〗^(3+)- reducing levels. In the PTIO⋅-scavenging assay, its IC50 value varied with pH. In the 〖Fe〗^(2+)-binding reaction, taxifolin was found to produce a green solution with two UV-visible absorbance peaks. At the same time, the possible toxicity of taxifolin to bmMSCs was measured by the CCK-8 assay (a newer version of the MTT assay). The results showed that taxifolin had no effect on proliferation and no toxic effect on normal bmMSCs (Li et al., 2017). Taxifolin could inhibit the oxidative stress and apoptosis of human umbilical vein endothelial cells (HUVECs) and THP-1 cells induced by Cr(VI), inhibit the activation of the NF-κB signal and downregulates the expression of cleaved caspase-1 and IL-1β in THP-1 cells, and prevent the adhesion of THP-1 cells to HUVECs by reducing the expression of ICAM-1 and VCAM-1 (Cao et al., 2020).

### 2.2 Anti-inflammatory activity

As a flavonoid, taxifolin has been found to have anti-inflammatory properties (Gupta et al., 1971). Wang et al. found that taxifolin inhibited the enhanced activity of NF-κB in cerebral ischemia-reperfusion injury rats and demonstrated that this is caused by taxifolin’s antioxidant action. Furthermore, taxifolin inhibits the infiltration of white blood cells and the expression of COX-2 and iNOS in cerebral ischemia-reperfusion injury, as well as the expression of Mac-1 and ICAM-1 (Wang et al., 2006). The role of taxifolin in modulating the inflammatory response in endotoxemia was investigated using Raw 264.7 cells and mice challenged with lipopolyssacharide (LPS) endotoxin. According to the results, taxifolin treatment significantly decreased the transcription of TNF-α, IFN-γ, IL-10 and TLR-4 in Raw 264.7 cells. Additionally, taxifolin induced AMPK/Nrf2/HO-1 signaling axis and enhanced Nrf2 expression and phosphorylation. As a result of pretreatment with taxifolin, mice were significantly less likely to die after being exposed to the bacterial endotoxins LPS for 10 days (Lei et al., 2020). In addition, Pan et al. investigated the effects of taxifolin on human mast cells (HMC-1), rat basophilic leukemia (RBL)-2H3, and bmMSCs. Specifically, they found that taxifolin inhibited the degranulation of bone marrow-derived mast cells, the production of leukotriene C4 (LTC4) and interleukin-6 (IL-6), and the expression of cyclooxygenase-2 (COX-2). Taxifolin can also inhibit the activation of RBL-2H3 and HMC-1 cells through Akt/IKK/NF-κB and MAPKs/cPLA2 signaling pathways (Pan et al., 2019). The results of these studies suggest that taxifolin could be a potential drug candidate for the treatment of allergic and inflammatory conditions.

### 2.3 Other pharmacological activities

In addition, taxifolin has demonstrated biological activities such as anti-Alzheimer activity (Sato et al., 2013a), antimicrobial activity (Cushnie and Lamb, 2005), anticancer activity (Lee et al., 2007), hepatoprotective activity (Akinmoladun et al., 2018), antiangiogenic activity (Haque et al., 2015), cardiovascular activity (Shu et al., 2019) and pulmonary activity (Jh et al., 2020) which make it an effective treatment for a variety of diseases.

## 3 Therapeutic effects of taxifolin

Numerous studies have been conducted on taxifolin to treat diseases between 2019 and 2022. An introduction to the therapeutic effects of taxifolin on the human body follows below.

### 3.1 Therapeutic effects of taxifolin in nervous system

#### 3.1.1 The effect of taxifolin on cerebral amyloid angiopathy and Alzheimer’s disease

There has been a great deal of research conducted in recent years regarding taxifolin’s ability to inhibit cerebral amyloid angiopathy (CAA) and Alzheimer’s disease (AD).

**FIGURE 2:**
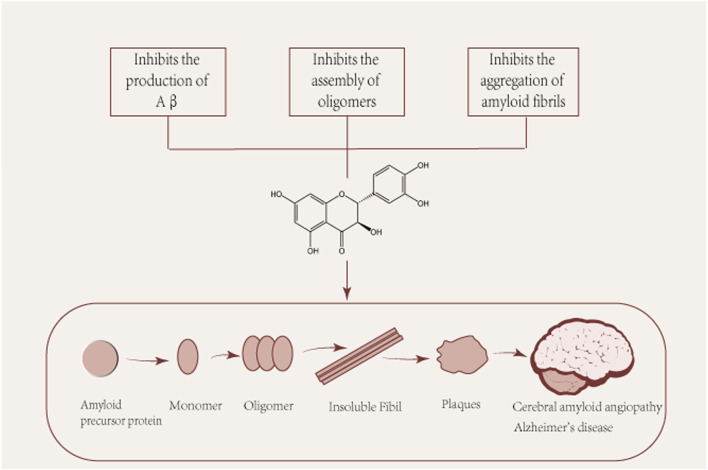
Pleiotropic beneficial effects of taxifolin in the formation of Aβ. Aβ: β-amyloid. Aβ is produced and undergoes various assembly forms, aggregating from monomers into oligomers and subsequently forming protofibrils and fibers, whose different forms play different roles in the pathogenesis of AD.

Several pathological studies have shown that more than 90% of patients with Alzheimer’s disease have CAA, suggesting a common pathogenic mechanism. CAA is characterized by the accumulation of β-amyloid (Aβ) in the walls of cerebral vessels, leading to complications such as intracerebral hemorrhage, convexity subarachnoid hemorrhage and cerebral microinfarcts (Saito et al., 2021). The pathological hallmark of Alzheimer’s disease is the deposition of Aβ in cerebellar vessels, age spots, and neurofibrillary tangles (Glenner and Wong, 1984; Glenner and Wong, 1984). Aβ protein is a common culprit in the pathogenesis of both ACC and Alzheimer’s disease and is the main subject of discussion in this section. Aβ is produced in neurons by amyloid precursor protein (APP) by β and γ secreting enzymes. Aβ is produced and undergoes various assembly forms, aggregating from monomers into oligomers and subsequently forming protofibrils and fibers, whose different forms play different roles in the pathogenesis of AD (Figure 2). Among them, Aβ oligomers are formed spontaneously by aggregation of Aβ proteins. Although senile plaques consist mainly of fibrous Aβ deposits, Aβ oligomers play a central role in the pathogenesis of AD, where they have been shown to cause a high degree of synaptic toxicity (Gaspar et al., 2010; Koffie et al., 2012; Savage et al., 2014; Yang et al., 2017; Hong et al., 2018) and their levels have also been shown to correlate with disease progression and severity of clinical symptoms (Fukumoto et al., 2010; Esparza et al., 2013; Tai et al., 2013). However, it is argued that taxifolin’s ability to cross the blood-brain barrier is too low to prevent it from functioning, which implies that taxifolin still needs further study (Yang et al., 2016).

##### 3.1.1.1 Taxifolin inhibits the production of Aβ

According to a mouse model of CAA, taxifolin inhibited the intracerebral production of amyloid-β through suppressing the ApoE-ERK1/2-amyloid-β precursor protein axis. At the same time, taxifolin suppressed inflammation, alleviated the accumulation of TREM2-expressing cells in the brain. Additionally, it reduced glutamate levels and oxidative tissue damage, as well as the level of active caspases in the brain, which is indicative of apoptotic cell death (Inoue et al., 2019).

In addition to this, BACE1 is another target of taxifolin to inhibit Aβ synthesis, which is the rate-limiting enzyme of the Aβ production pathway, and APP is cleaved by BACE1 to produce Aβ (Zhou et al., 2011). According to S et al., a QSAR model was developed to identify natural compounds as potent BACE1 inhibitors, and the ability of these compounds to inhibit BACE1 activity was validated. As a result of the screening of this model, taxifolin showed moderate antioxidant activity in addition to excellent BACE1 inhibitory activity (Sucharita et al., 2020).

##### 3.1.1.2 Taxifolin inhibits the assembly of oligomers

It was also found that taxifolin prevented the assembly of amyloid β oligomers. Saito et al. applied taxifolin to Tg-SwDI mice and the filter capture assay and ELISA showed that the brain homogenate of Tg-SwDI mice showed a significant reduction in the level of Aβ oligomers *in vivo* after taxifolin treatment (Saito et al., 2017).

##### 3.1.1.3 Taxifolin inhibits the aggregation of amyloid fibrils

**FIGURE 3:**
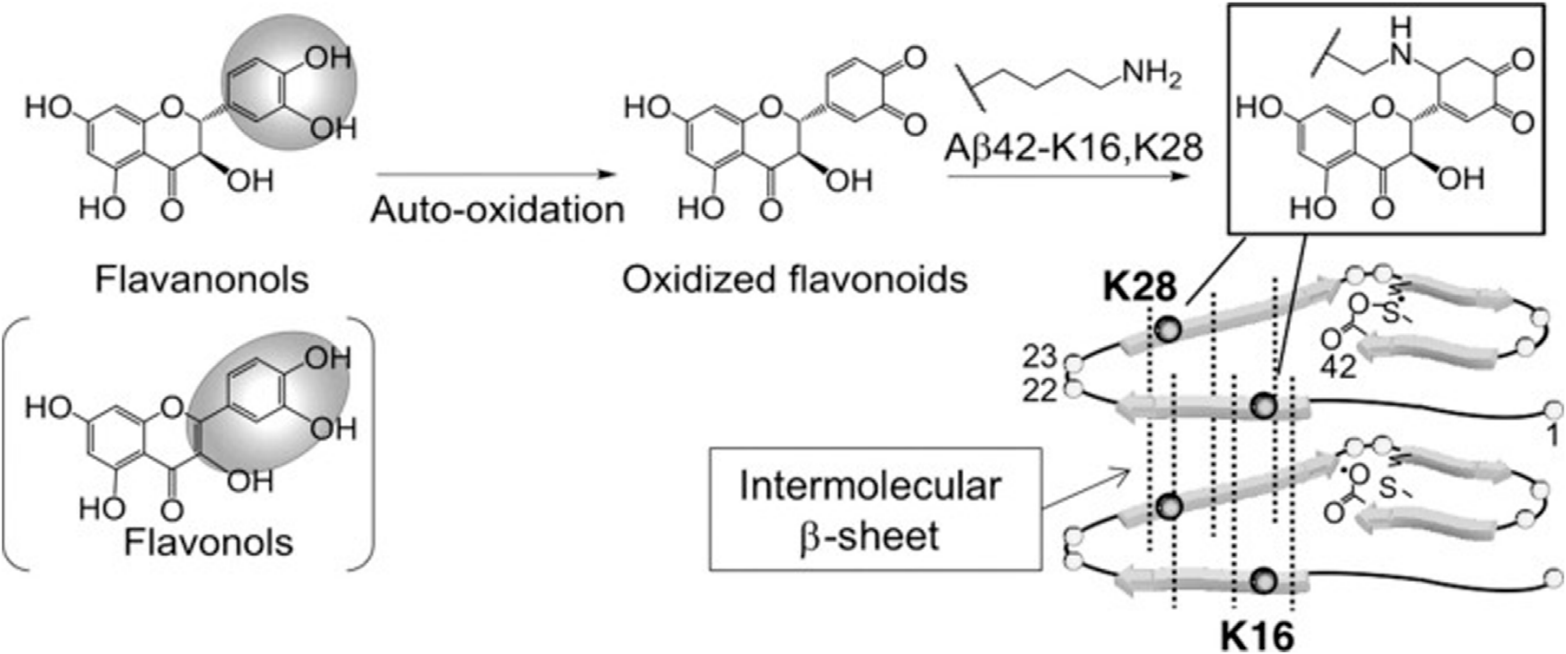
Site-specific inhibitory mechanisms of Aβ42 aggregation by catechol-type flavonoids (Sato et al., 2013a). Reproduced with permission from Sato et al., 2013b. Catechol-type flavanonols (e.g., (+)-taxifolin) or flavonols (e.g., quercetin) were oxidized to form corresponding o-quinones on B-ring, followed by the formation of adducts by Lys16 and Lys28 of Aβ42. Because Lys16 and Lys28 are incorporated in the intermolecular β-sheet region (Murakami et al., 2005), Aβ42 aggregates would be destabilized by the adduct formation.

Sato et al. performed a structure-activity relationship study of (+)-taxifolin showing that the 3′,4′-dihydroxyl groups were critical for the ability of taxifolin to resist aggregation of the 42-residue amyloid β-protein (Aβ42), while the 7-hydroxyl group and the stereochemistry of the 2 and 3 positions were not relevant (Sato et al., 2013a). Sato et al. also proposed a mechanism for the specific inhibition of Aβ42 aggregation by (+)-taxifolin, and suggested that taxifolin specifically inhibits Aβ42 aggregation by targeting Lys residues (Sato et al., 2013b) (Figure 3). In a subsequent study, Ginex et al. further demonstrated that the oxidized form of (+)-taxifolin binds to the edge-delineated hydrophobic groove in amyloid fibrils defined by Lys16 and Glu22 residues, forming covalent adducts that interfere with amyloid fibril aggregation (Ginex et al., 2018). This specific inhibitory mechanism may explain the enhanced anti-aggregation activity of oxidized flavonoids compared with fresh compounds, offering hope for the development of disease-modifying therapies.

#### 3.1.2 The effect of taxifolin on Parkinson’s disease

Parkinson’s disease (PD) is the second most common age-related neurodegenerative disease, after Alzheimer’s disease, with a prevalence of more than 1.6% among individuals over 65 years of age (Stoker and Greenland, 2018). In the study, Akinmoladun C Afolabi and others administered 1.5 mg/kg rotenone to male Wistar rats for 10 days followed by 3 days of taxifolin treatment for 3 days, assessing rat brain striatal redox stress and neurochemical dysfunction markers by spectrophotometry, as well as histological changes in the striatum. A significant inhibitory effect of taxifolin was observed in the results is that taxifolin inhibited the upregulation of IL-1β, NF-κB and IκKB expression (*p* < 0.05) in the striatum of parkinsonian rats, weakened the disruption of the striatum dopaminergic and cholinergic systems caused by rotenone, controlled glutamate metabolism and mitochondrial complex dysfunction, so taxifolin could improve the neurobehavioral and dysfunctional disorders of rat Parkinson’s disease caused by rotenone (Akinmoladun et al., 2022).

#### 3.1.3 The effect of taxifolin on glioblastoma multiforme

The most common primary malignant brain tumor is glioblastoma multiforme (GBM), with a poor prognosis. A silicon analysis and *in vitro* experiments have demonstrated that taxifolin inhibits mTOR/PI3K, promotes autophagy, and decreases lipid synthesis in GBM. Taxifolin has been shown to inhibit GBM in mice in vivo experiments. Taxifolin is considered to be a valuable drug for the treatment of GBM by W et al. (Yao et al., 2021).

#### 3.1.4 The effect of taxifolin on glioblastoma multiforme

Despite its protective effects on neurotoxins, taxifolin is unclear in terms of its mechanism of action. Chlorpyrifos (CPF) is an organophosphorus insecticide that can damage the central nervous system of children after exposure. Zhang et al. demonstrated that taxifolin treatment protected against CPF-induced neurotoxicity by downregulating ROS, TNF-α, IFN-γ and p62 levels and increasing LC3 II levels, thereby improving BV2 cell activity and viability (Zhang C. et al., 2019). As a result of the oxidative stress caused by cisplatin on rats’ optic nerves, these changes result in significant histopathological damage. However, histopathological examination in the group treated with taxifolin is close to normal, except for mild neuropleural swelling. Therefore, taxifolin has been shown to be used to prevent oxidative damage to the optic nerve caused by cisplatin (Ahiskali et al., 2021).

As reported by Gunesch et al., 7-O-ester hybrids of flavonoid taxifolin with phenolic acids cinnamic and ferulic acid, namely, 7-O-cinnamoyltaxifolin and 7-O-feruloyltaxifolin, have been synthesized. In the murine hippocampal neuron HT22 cell model, these compounds demonstrated neuroprotective effects against oxytocin, ferroptosis, and ATP depletion. Furthermore, 7-O-cinnamoyltaxifolin and 7-O-feruloyltaxifolin were shown to reduce LPS-induced neuroinflammation in BV-2 microglia cells as measured by NO, IL6 and TNFα levels. It has been shown that the treatment of the 7-O-cinnamoyltaxifolin and 7-O-feruloyltaxifolin alleviated memory deficits in mice models of AD, in which oligomeric A25-35 peptides were injected into the mouse brain to induce neurotoxicity (Gunesch et al., 2020).

### 3.2 Therapeutic effects of taxifolin in immune system

#### 3.2.1 Effect of taxifolin on viruses

##### 3.2.1.1 Effect of taxifolin on SARS-CoV-2

On the one hand, the role of taxifolin in targeting SARS-CoV-2’s main protease (Mpro) has been debated among scholars. Gogoi et al. docked 44 citrus flavonoids to the highly conserved Mpro of SARS-CoV-2 and predicted activity (IC50) based on 3D-QSAR analysis after drug likeness and toxicity parameters. As a result, taxifolin has the lowest predicted IC50, which indicates that it may be a potential inhibitor of SARS-CoV-2’s primary protease (Gogoi et al., 2021). Under the premise of molecular docking with SARS-CoV-2 Mpro, Al-Karmalawy et al. tested IC50 and CC50 of five flavonoid compounds through *in vitro* experiments, and took the co-crystallized inhibitor of SARS-CoV-2 Mpro (α-ketoamide inhibitor (KI), IC50 = 66.72 μg/mL) as a reference standard. The IC50 value of taxifolin is greater than 78.690 mg/mL, which is greater than the α-ketoamide inhibitor, and taxifolin is believed to be the best flavonoid for inhibiting SARS-CoV-2 (Al-Karmalawy et al., 2021). There is a difference between the conclusions reached by A and B, and both have their own basis. According to A, a scientific prediction can be made using simulated 3D-QSAR analysis, and according to B, a conclusion can be drawn from *in vitro* tests. Further investigation is needed to determine the effect of taxifolin on the Mpro of SARS-CoV-2.

**FIGURE 4:**
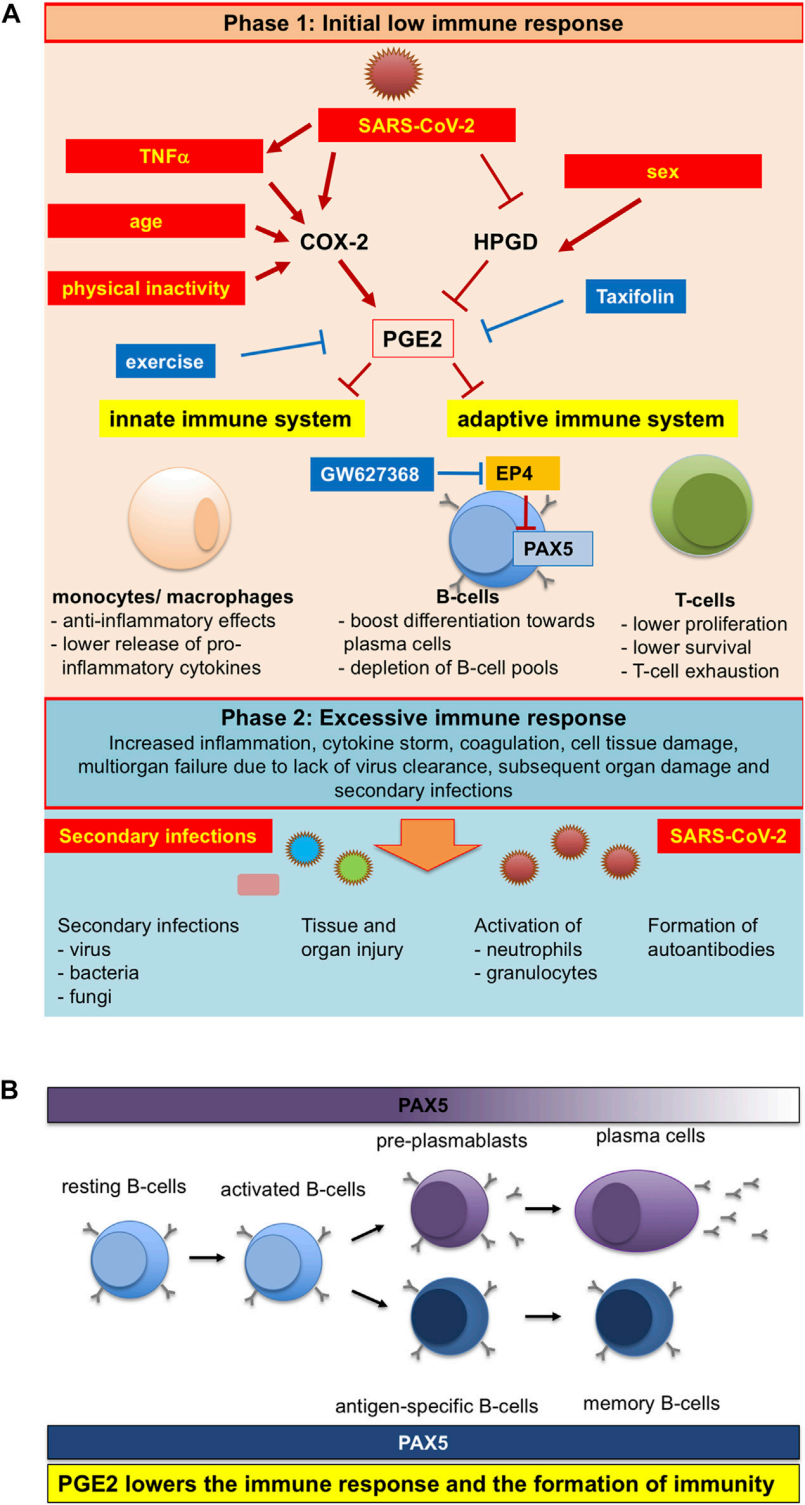
Schematic representation of pleiotropicinfluences of SARS-CoV-2 infection, physical activity and age on PGE2 levels and the ensuing altered immuneresponse (Ricke-Hoch et al., 2021). Reproduced with permission from Ricke-Hoch et al., 2021. **(A)** SARS-CoV-2 infection, physical inactivity, sex and older age are modulators of PGE2 synthesis and degradation, which are also risk factors for more severe COVID-19 disease courses. **(B)**PGE2 lowers the immune response and the formation of immunity.

On the other hand, taxifolin can reduce SARS-CoV-2-induced PGE2 production. In COVID-19 patients, serum levels of PGE2 were significantly elevated and positively correlated with disease severity. As a consequence, the levels of PGE2 in patient serum decreased the expression of PAX5 in human pre-B cells. PAX5 is a master regulator of B cell survival, proliferation, and differentiation, and a master regulator of long-lived memory B cells. Thus, taxifolin treatment can prevent serious disease processes (Ricke-Hoch et al., 2021) (Figure 4).

In 2021, a clinical trial exploring the effect of taxifolin as a dietary supplement on the recovery period after COVID-19 pneumonia began in Russia. During this trial, the primary objective is to monitor the effects of taxifolin aqua therapy on the indicators of respiratory function, arterial wall condition and contractile function of the myocardium, in addition to assessing the effect of Taxifolin Aqua therapy on markers of biological age and patient quality of life (National Library of Medicine, ClinicalTrials.gov, 2023).

##### 3.2.1.2 Effect of taxifolin on HIV

HIV infection affects millions of people worldwide, and combined antiretroviral therapy (cART) has greatly improved the quality of life for people with HIV25; however, current cART faces a number of challenges regarding its lifelong persistence (Palmisano and Vella, 2011). Consequently, there is a critical need for the development of new anti-HIV drugs and effective microbicides, of which taxifolin may be one. The IC50 of taxifolin extracted from Cassia abbreviata is 49.04μM, exhibiting strong anti-HIV-1 activity (Yang et al., 2021). Additionally, six substances, including taxifolin isolated from the alcohol crude extract of C. abbreviata root and bark, can block HIV-1 entry into cells (Zheng et al., 2021).

#### 3.2.2 Effect of taxifolin on bacteria

Phenolic Flavonoids derived from plants possess antibacterial activities (Cowan, 1999).The mechanisms of bacterial inhibition by phenolic flavonoids are: inhibition of cell plasma membrane function, nucleic acid synthesis and energy metabolism (Cushnie and Lamb, 2005). Taxifolin is also a flavonoid with strong antibacterial properties against *Staphylococcus aureus*, *Escherichia coli*, *Shigella* and *Salmonella* (Yang et al., 2019).

**FIGURE 5:**
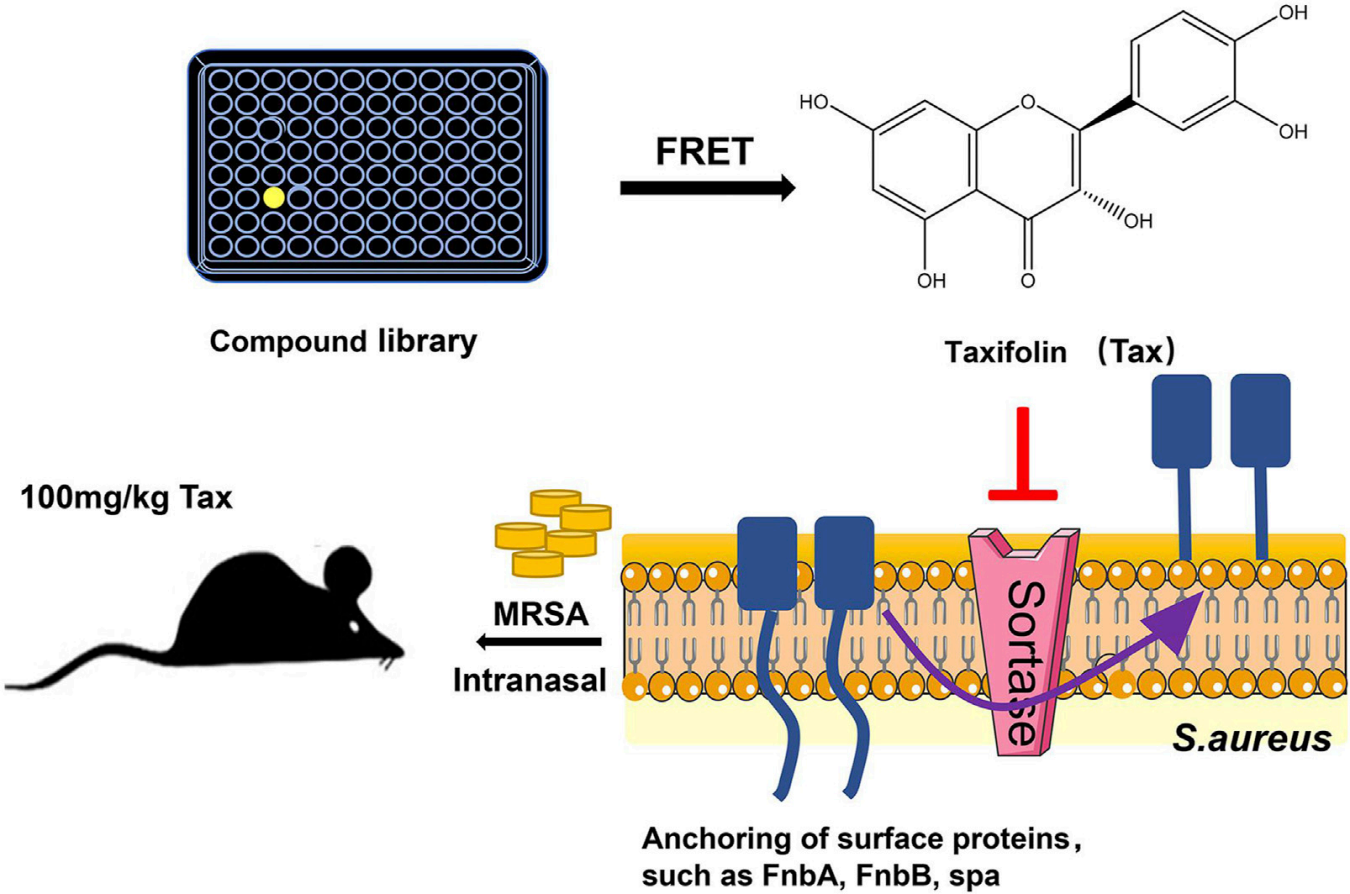
Experimental flow chart (Wang et al., 2021). Reproduced with permission from Wang et al., 2021. Taxifolin inhibits biofilm formation by inhibiting the anchoring of surface proteins required for the biofilm adhesion phase through inhibition of cysteine transpeptidase sortase A, thereby protecting mice from MRSA-induced pneumonia at lethal doses.

New developments have been made in the mechanism of taxifolin against *Staphylococcus aureus* infection, proving that taxifolin can also prevent the development of drug-resistant strains of *Staphylococcus aureus* during infection, and *in vivo* experiments have demonstrated that taxifolin protects mice from the deadly effects of methicillin-resistant *Staphylococcus aureus* (MRSA)-induced pneumonia. *Staphylococcus aureus*’ cysteine transpeptidase A (SrtA) mediates the anchoring of proteins on their surfaces. Inhibitors of SrtA do not interfere with bacterial growth, but can weaken the virulence of bacteria (Suree et al., 2007; Hou et al., 2018).As a result, they are capable of preventing colonization and invasive disease caused by *Staphylococcus aureus*, but have a lower risk of bacterial resistance (Hou et al., 2018). Due to its ability to bind to the Asp-170 and Gln-172 sites of SrtA, taxifolin is considered a potent SrtA inhibitor. *In vitro*, taxifolin can reduce adhesion, anchoring and hinder biofilm formation of *Staphylococcus aureus*. *In vivo*, taxifolin protects mice from lethal doses of MRSA-induced pneumonia, significantly improving their survival rates and reducing the number of viable *Staphylococcus aureus* in lung tissue (Wang et al., 2021) (Figure 5). Taxifolin inhibits SRTA activity without interfering with the growth of bacteria, preventing the development of drug-resistant strains, which has become an increasingly significant global problem today. As a pioneer compound for new agents against *Staphylococcus aureus* infection, it has the potential to become a new drug for treating MRSA-induced pneumonia, as well.

According to previous studies, taxifolin showed a high level of activity against the Gram-positive bacterium *Staphylococcus aureus in vitro*, while the Gram-negative bacteria *Pseudomonas aeruginosa* and the fungus *Candida* albicans are not sensitive to taxifolin. In the study of the antibacterial efficacy of plant polyphenols as topical drugs *in vivo*, Shevelev et al. found that both were very sensitive to taxifolin *in vivo*, and it is believed that taxifolin, in conjunction with antibiotics, can be used to prevent suppuration in early stages of trauma treatment (Shevelev et al., 2020).

### 3.3 Therapeutic effects of taxifolin in digestive system

#### 3.3.1 Effect of taxifolin on stomach diseases

**FIGURE 6:**
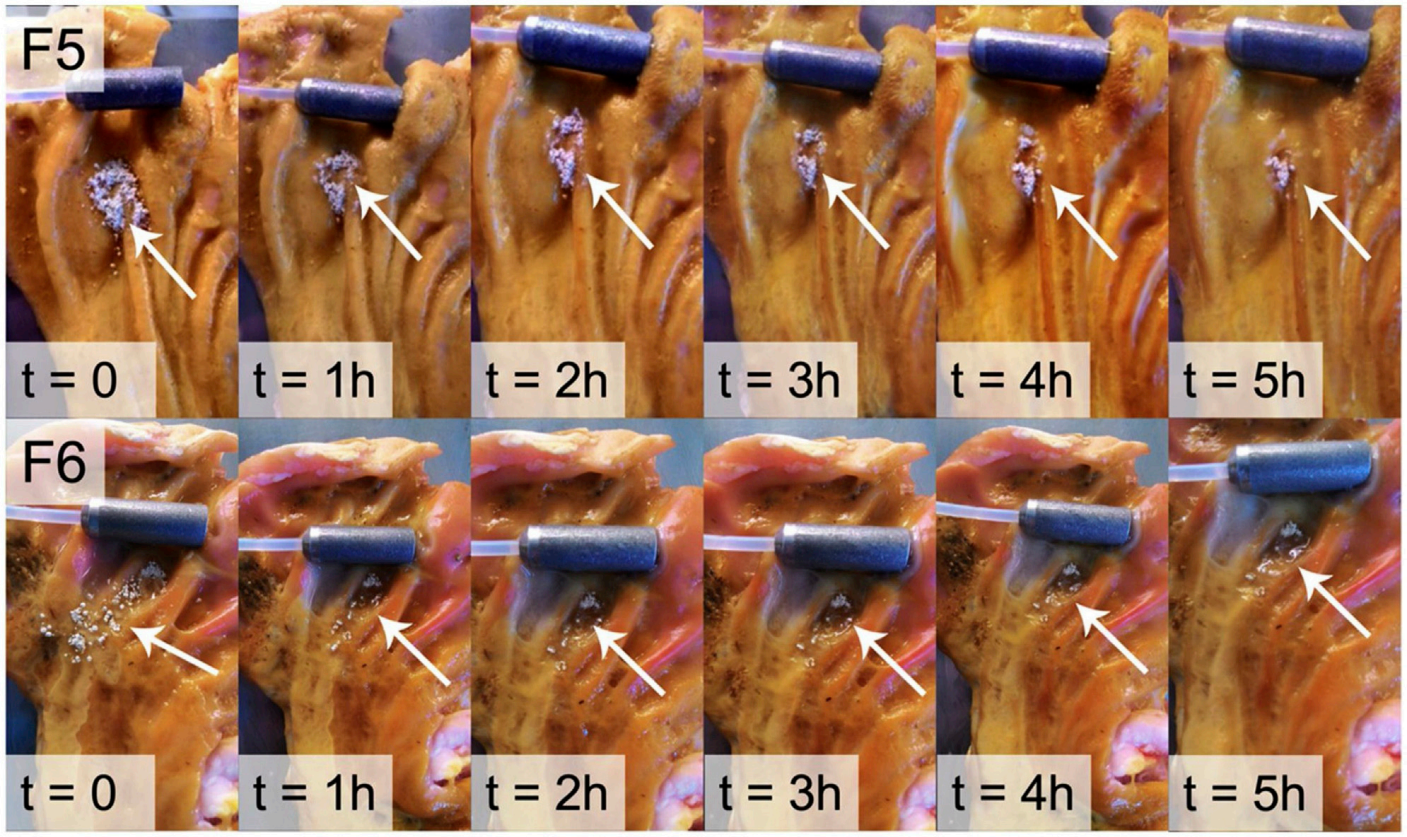
*Ex vivo* mucoadhesion duration of F5 and F6 determined on pig gastric mucosa (Stenger Moura et al., 2019). Reproduced with permission from Stenger Moura et al., 2019. The adhesion time of the F5 and F6 microparticles was monitored for 5 h. Both formulations adhered to the gastric mucosa during this period.

According to Schlickmann et al., taxifolin has similar gastroprotective effects to omeprazole, with 41% proton pump inhibition. However, the mechanism of action remains unclear (Schlickmann et al., 2015). Taxifolin has low water solubility, is unstable in alkaline media, and is degraded by the intestine. Based on chitosan, HPMC and mesoporous silica materials, Stenger Moura et al. developed a taxifolin mucoadhesive formulation to treat gastric ulcers. In this study, the optimized microparticles were able to release taxifolin and adhere to porcine stomach mucosa for a period of 5 hours (Stenger Moura et al., 2019) (Figure 6). On the basis of the previous study, Stenger Moura et al. has applied gastro-mucoadhesive microparticles containing taxifolin (MPTax) to rats with acetic acid-induced gastric ulcers. As a result of the study, MPTax significantly reduced the area of gastric ulcers compared to the controgroup. It also reduced myeloperoxide levels, increased stomach mucin levels, decreased the activity of myeloperoxidase, and increased glutathione levels at the ulcer site. In addition, MPTax demonstrated a reversible interaction with H+/K + -ATPase *in silico*, confirming its anti-H. pylori effect. (MIC = 625 μg/mL). C et al. believe that the oral treatment of acidic digestive diseases may offer significant promise (Stenger Moura et al., 2021a).

#### 3.3.2 Effect of taxifolin on intestines diseases

Wan et al. demonstrated that taxifolin can effectively alleviate colitis induced by dextran sodium sulfate (DSS), and that colon tissue with disease activity index (DAI), colon length, and histopathological scores of colon tissue have all been restored to some extent (Wan et al., 2021).

Importantly, changes in the diversity and composition of the intestinal flora may change the homeostasis of the body, thus affecting the health of the elderly and the susceptibility to diseases. In addition, the brain–gut axis plays an important role in the connection between the brain and the intestine, while intestinal flora can directly or indirectly affect the brain–gut axis and then affect brain function (Rinninella et al., 2019; Cryan et al., 2020).

Liu et al. established an aging model by intraperitoneal injection of D-galactose in mice, and after treatment with taxifolin, spatial learning and memory impairment were significantly restored, histopathological damage and structural disorders in the hippocampus region of mouse brain tissue were reversed, and Nrf2-mediated oxidative stress was also inhibited. At the same time, the composition of the intestinal flora and the abundance of beneficial bacteria have changed significantly. Liu et al. recognized that taxifolin delayed the D-galactose-induced aging process by inhibiting Nrf2-mediated oxidative stress and modulating the intestinal microbiota of mice, which provides the possibility of prevention and treatment for aging and metabolic disorders through the potential food health factors (Liu et al., 2021).

Su et al. showed that taxifolin inhibited Proteobacteria from blooming and improved the dysbiosis of intestinal microbiota while also improving obesity symptoms, liver steatosis, and lipid peroxidation in C57BL/6J mice fed high-fat diets (Su et al., 2022).

#### 3.3.3 Effect of taxifolin on liver

The liver plays an important role in energy metabolism and the biotransformation of foreign substances, which makes it vulnerable to toxicity or damage (Park et al., 2005; Katarey and Verma, 2016). Liver damage caused by certain foreign bodies is a recognized toxicological problem known as hepatotoxicity. Silymarin is a widely used hepatoprotective drug in which taxifolin is found to be an essential component (Legalon^®^). Due to its excellent antioxidant and anti-inflammatory properties, taxifolin has detoxification effects on a variety of toxic substances and has a strong liver protection effect. The presence of double bonds at the position of C2-C3 in the taxifolin C ring is essential for hepatoprotective activity (Akinmoladun et al., 2018).

Pazopanib is a tyrosine kinase inhibitor commonly used to treat metastatic renal cell cancer and advanced soft tissue sarcoma, but can cause varying degrees of liver toxicity. To address this problem, Akagunduz et al. explored the effects of taxifolin on the liver toxicity caused by pazopanib. The results showed that after prophylactic use of taxfolin, Malondialdehyde (MDA), total glutathione (tGSH), total oxidant status (TOS), and total antioxidant status (TAS) levels were improved; Liver tissue damage, including bleeding, edema degeneration, and necrosis, was also reduced. Administration of taxifolin prior to pazopanib has been shown to be hepatotoxic in rats, significantly improving degenerative changes caused by pazopanib. Taxifolin is expected to compensate for the deficiencies of pazopanib, reduce its adverse effects, and bring good news to more patients with metastatic renal cell carcinoma and advanced soft tissue sarcoma (Akagunduz et al., 2021).

Iron is essential for many physiological processes, but excess iron can cause liver damage. The chelating complex of taxifolin with iron showed stronger antioxidant activity than the unchelated parent compound, a property that enhances the potential protective effect of taxifolin against liver tissue damage caused by iron overload (Moridani et al., 2003; Grazul and Budzisz, 2009). The effect of taxifolin on hepatocyte damage induced by iron excess was explored in rats as a mammalian model, indicating that taxifolin reduced the iron content in the liver through its biological characteristics of anti-inflammatory, antioxidant and iron chelating activity, effectively alleviated iron-induced histopathological aberrations, enhanced the regenerative ability of the liver, and improved the survival rate of liver cells (Salama and Kabel, 2020).

The effect of taxifolin on hepatotoxicity caused by acetaminophen (APAP) was studied. Hu et al. took C57 mice and L-02 cells as the study object, continuously administered taxifolin for 7 days, and APAP was administered on the last day to establish a model of acute liver injury. Pharmacodynamic, pharmacological, and metabolomics analyses were then evaluated. The results suggest that taxifolin prevents APAP-induced liver injury by inhibiting the activation of APAP metabolism mediated by CYP450 enzymes and regulating the expression of glutathione metabolism and related antioxidant signals (Hu et al., 2019).

Yang et al. explored the liver protection mechanism of taxifolin on CCl4-induced acute liver injury mice, and the results showed that taxifolin significantly limited the increase of sALT and sAST, reduced the liver lesion range and vacuole formation in mice, and also significantly reduced neutrophil infiltration and necrosis. In addition, SOD, GPx, and GRd activity increased and MDA levels decreased. Yang et al. believe that taxifolin may increase antioxidant enzyme activity by exerting antioxidant properties, which in turn causes MDA levels to decrease, exerting its protective effect on liver tissue (Yang et al., 2019).

Taxifolin not only has therapeutic potential in liver damage caused by foreign bodies, but also has outstanding progress in the treatment of the mechanism of chronic fatty liver caused by the combination of obesity and overeating alcohol. Taxifolin has been shown to have therapeutic potential for both alcoholic hepatitis and fatty liver, however, how taxifolin regulates chronic hepatic fatty liver caused by the combination of obesity and overeating alcohol remains ununderstood. Recently, Zhan et al. established *in vivo* models and *in vitro* models of HepG2 cells respectively in order to explore the mechanism of action of taxifolin on chronic steatohepatitis induced by high-fat diet (HFD) feeding plus acute ethanol binge. The results show that taxifolin can effectively inhibit the expression of SREBP1 and upregulate PPARγ levels, and also inhibit the expression of P2X7R and IL-1. In addition, taxifolin reduces the activation expression of caspase-1 and thus inhibits the recruitment of macrophages and neutrophils, as well as the inflammatory response. Taxifolin therefore has therapeutic potential as an intervention in alcohol- and hyperlipidaemia-induced steatohepatitis and to prevent nonalcoholic fatty liver degeneration targeting caspase-1 (Zhan et al., 2021).

In addition, in the compound activity data from the liver protection assay against hepatitis C virus infection, taxifolin showed strong activity in four out of five assays, with liver protection observed at lower doses compared to the other compounds, and plateaued after reaching a nadir at low doses. Taxifolin was demonstrated to inhibit viral infection, virus-induced oxidative stress, NF-κB-dependent transcription, and TCR-mediated proliferation (Polyak et al., 2010).

### 3.4 Therapeutic effects of taxifolin in cardiovascula

There are a variety of cardiovascular diseases (CVD) that relate to the heart or blood vessels, such as heart failure, hypertension, coronary artery disease, and atherosclerosis (Middleton et al., 2000). Various experimental and epidemiological studies have demonstrated that the consumption of foods rich in flavonoids is associated with a reduced risk of cardiovascular disease (Stenger Moura et al., 2019). It has been suggested that taxifolin may play an influential role in the prevention and treatment of cardiovascular disease. According to one study, taxifolin can inhibit the synthesis of cholesterol in HepG2 cells and also reduce liver lipid synthesis by decreasing and increasing apoB and apoA-I secretion. Furthermore, taxifolin significantly inhibits the esterification of cholesterol in cells, triacylglycerol and phospholipid synthesis (Theriault et al., 2000). In addition, taxifolin has a certain protective effect on ischemia-reperfusion injury. Recent studies have shown that the protective effect of taxifolin against ischemia-reperfusion injury may be accomplished by activating the PI3K/Akt pathway (Shu et al., 2019).

**FIGURE 7:**
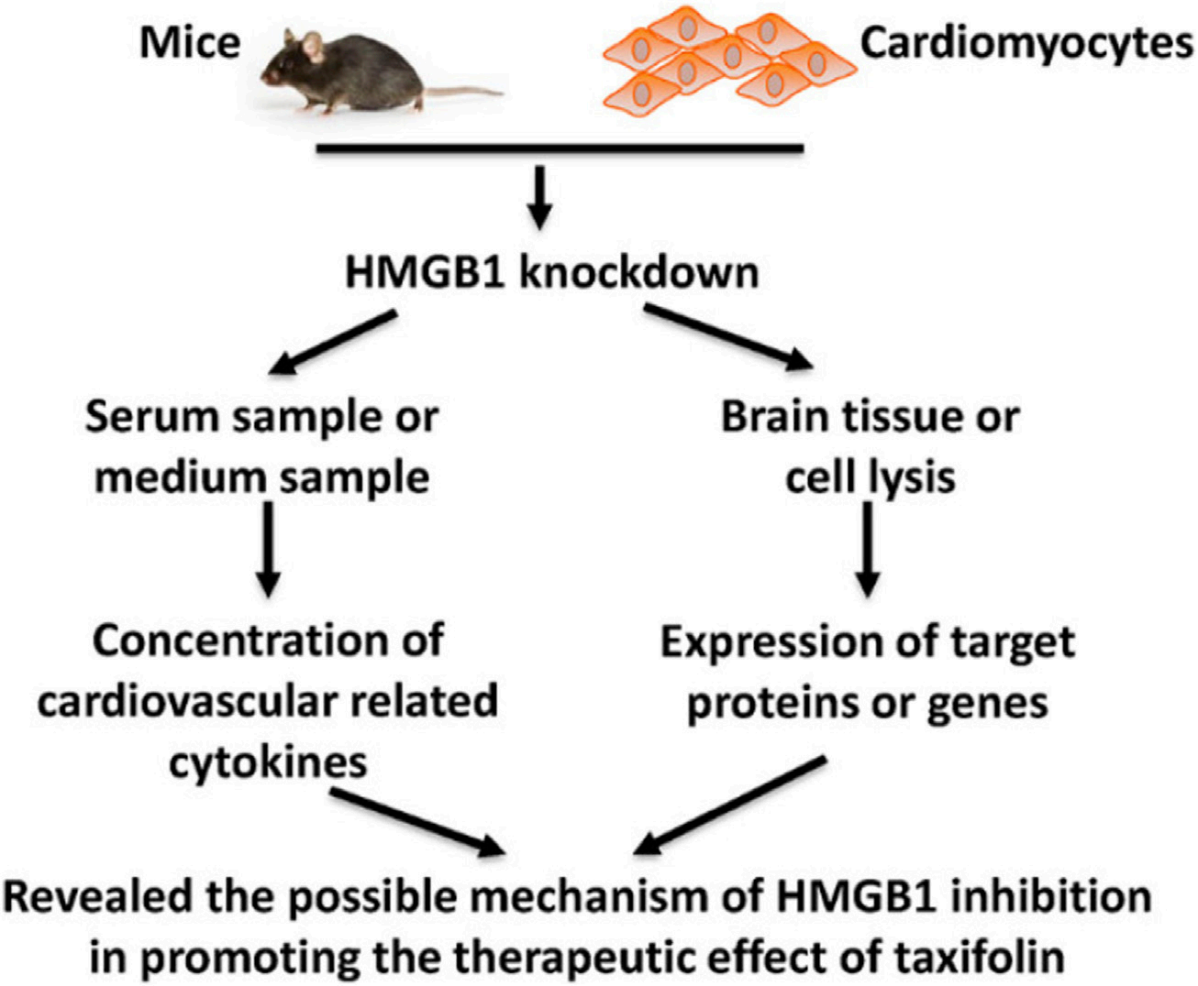
Schematic figure of present study (Feng et al., 2021). Reproduced with permission from Feng et al., 2021. A cellular model of HMGB1 overexpression and knockdown was established in H9c2 cells, and HMGB1 was found to enhance the protective effect of taxifolin on these cells, probably through the PI3K/AKT/mTOR/ERK signaling pathway.

It has been found that inhibiting HMGB1 can enhance the cardiovascular effects of taxifolin through the PI3K/AKT/mTOR signaling pathway, which may prove to be a new therapeutic strategy for cardiovascular disease. Taxifolin can reduce the expression of HIF-1α while increasing the expression of eNOS through the mediation of the PI3K/AKT/mTOR signaling pathway. Additionally, Taxifolin is capable of increasing the expression of VEGF-α, TGF-β and FGF21. A similar trend was observed in mice that were knocked down for HMGB1 (Feng et al., 2021) (Figure 7).

A plasticizer known as di-(2-ethyl hexyl) phthalate (DEHP) may cause cardiovascular disease in animals, although its specific mechanism of action is unknown. Y et al. conducted experiments to explore the mechanism of cardiomyocyte necrosis caused by DEHP and the effect of taxifolin on this process. The results showed that the concentration of Ca2+ in cardiomyocyte cytoplasm decreased, the necrosis rate of cardiomyocytes decreased, and the expression of genes related to the necrotic calcium pathway decreased in the DEHP + taxifolin dermatogen group compared to the DEHP group. DEHP induces cardiomyocyte necrosis by triggering calcium overload, and TAX can alleviate this condition (Yingying et al., 2020).

Cai et al. investigated the specific molecular mechanisms of DEHP-induced cardiomyocyte masturbation and the protective effect of taxifolin. The results showed that DEHP induced cardiac hypertrophy by activating the IL-6/JAK/STAT3 signaling pathway, causing glucose metabolism disorders and extracorporeal mitochondrial dysfunction, which could be significantly improved by taxifolin (Cai et al., 2019).

### 3.5 Therapeutic effects of taxifolin in respiratory system

There is growing evidence that taxifolin plays an instrumental role in respiratory disease. An effective treatment for acute lung injury is provided by taxifolin, which inhibits inflammation caused by acute lung injury. Taxifolin can induce the expression of miR-132-3p, whereas FOXO3 is the target of miR-132-3p. It is possible that increased expression of miR-132-3p can inhibit the expression of FOXO3, thereby suppressing the NF-κB pathway activated by FOXO3, weakening the LPS-induced inflammatory response and apoptosis, thereby reducing inflammation caused by acute lung injury (Jh et al., 2020).

Furthermore, taxifolin can also reduce lung damage caused by benzo [a]pyrene and cisplatin. Taxifolin improves lung damage caused by benzo [a]pyrene, an environmental pollutant and mutagen widely found in cigarette smoke, car exhaust particles, and other sources. Benzo [a]pyrene reduced the expression of mouse ROS-sensitive factor Nrf2 and its downstream targets NQO1, HO-1, SOD, while prophylactic administration of male Swiss albinmice with taxifolin could make Nrf2 and its downstream target expression levels high (Islam et al., 2021). For cisplatin-induced pulmonary oxidative damage in male albino Wistar rats, taxifolin gives full play to its antioxidant properties, providing protection against oxidative stress in the lungs by inhibiting the increase in oxidant parameters and the reduction of antioxidants (Unver et al., 2019).

Taxifolin is expected to become a promising drug for the treatment of asthma. There is increasing evidence that Matrix metalloproteinase-10 (MMP-10) is involved in the inflammatory response (Toni et al., 2013). In asthma biopsies, MMP-10 expression was elevated in epithelial cells as well as in subepithelial inflammatory cells and resident cells (Kuo et al., 2015). Airway remodeling and inflammation are associated with submucosal eosinophilia, in which MMP-10 may have an influential role (Kuo et al., 2019). Taxifolin inhibits the expression of MMP-10 by blocking the Wnt/β-catenin pathway, improving inflammatory damage to human bronchial epithelial cells (Chen et al., 2022).

It is not known whether taxifolin provides protection against iron poisoning caused by cigarette smoke and its mechanisms. Both *in vivo* and *in vitro*, Liu et al. demonstrated that taxifolin significantly reversed cigarette smoke-induced ferroptosis through Nrf2-dependent signaling (Liu et al., 2022).

The administration of a compound antioxidant preparation containing taxifolin to rats exposed to coal-rock dust for an extended period of time demonstrated that taxifolin compensated for disturbances in the redox balance in the lung tissues, prevented dust granulomas from forming, and reduced the severity of degenerative changes in the bronchopulmonary area (Zhukova et al., 2020).

### 3.6 Therapeutic effects of taxifolin on cancers

Several natural products have proved to be potent sources of anti-cancer drugs, as cancer is one of the leading causes of death worldwide (Butler, 2004; Ghosh et al., 2014). As a result of the continuous exploration of traditional Chinese medicine, medicinal plants have emerged as a unique treatment strategy for cancer, primarily due to their accessibility, low toxicity, and well tolerated nature (Takebe et al., 2011; Thakor et al., 2016). Natural bioactive compounds, especially flavonoids, have a significant impact on the treatment of cancer. Taxifolin exhibits significant anticancer activity, and there are mild or no side effects on normal healthy cells (Das et al., 2021). Taxifolin regulates genes associated with cancer, including those involved in hepatic detoxification, antioxidation, cell cycle, and cell growth, according to Lee et al. DNA microarrays from cancer DNA microarrays are used to demonstrate this association (Lee et al., 2007). Taxifolin is an antagonist of epidermal growth factor receptors and PI3K receptors. It has demonstrated many chemotherapeutic activities in cancer model systems, including antiproliferative, antiangiogenic, stemness and EMT regulation, among others.

It is pertinent to note, however, that taxifolin’s anticancer activity is not universally positive, and there are ambiguous and even conflicting results. ZEB2 protein is a transcription factor that plays an instrumental role in the epithelial/mesenchymal transition (EMT) (Vandewalle et al., 2009; Gonzalez and Medici, 2014). After treatment with taxifolin, the ZEB2 protein was upregulated in a dose-dependent manner. However, this regulation did not lead to an epithelial/mesenchymal transition. By inhibiting Akt phosphorylation, taxifolin reduced ZEB2 signaling, which could trigger cancer. Due to non-specific effects on cells, Z et al. suggest that taxifolin’s biological activity may have vague or even contradictory results (Z et al., 2021).

Taxifolin has not been shown to have an anti-tumor effect on gastric cancer in the studies conducted to date. To investigate the effect and mechanism of taxifolin on gastric cancer, Xie et al. treated AGS and NCI N87 cells with taxifolin. Viability and proliferation of cells are determined using Cell Counting Kit 8 and colony formation assays, migration and invasion capacity are determined by wound healing and Transwell assays, and protein expression is assessed using Western blots *in vitro* and *in vivo*. The results showed that taxifolin significantly inhibited the survival, proliferation, migration, invasion and tumor growth of gastric cancer through the aryl hydrocarbon receptor (AhR)/cytochrome P450 1A1 (CYP1A1) signaling pathway. Taxifolin may prove to be a potential treatment strategy for stomach cancer (Xie et al., 2021).

It is also unknown how taxifolin contributes to the treatment of highly aggressive breast cancer and what the underlying mechanisms are. According to Li et al., taxifolin inhibits proliferation, migration, and invasion of aggressive breast cancer cells and exhibits dose dependence. Furthermore, taxifolin significantly inhibited the growth of primary tumors and reduced lung metastases of breast cancer in a 4T1 xenograft mouse model. However, excessive expression of adenovirus to β-catenin diminishes these beneficial effects of taxifolin. Taxifolin is expected to be used as a promising drug for the clinical treatment of highly aggressive breast cancer (Li et al., 2019).

The expression of SOS1, a key regulator of the Ras pathway, is highly elevated in African American (AA) breast cancer patients. Taxifolin inhibited signal transduction of SOS1 by blocking the interaction between SOS1 and Grb2, demonstrating that taxifolin could be effective in combating SOS1-driven tumor progression, demonstrating the potential utility of the compound as a treatment for patients with AA-type breast cancer (Xing et al., 2021).

Taxifolin has the potential to become a new drug for the treatment of liver cancer. Liver cancer is the result of structural abnormalities in the blood vessels of the liver, and this angiogenesis is driven by the overexpression of hypoxia-inducible factor 1-α (Hif1-α) and vascular endothelial growth factor (VEGF), in addition, protein kinase B (Akt) are also compromised in liver cancer. A et al. confirmed that taxifolin showed positive docking scores with Hif1-α, VEGF and Akt through molecular docking experiments, and taxifolin could also directly affect the expression levels of the three. *In vitro* experiments showed that treatment with taxifolin could induce apoptosis in HepG2 and Huh7 cell lines. The potential of taxifolin in the treatment of liver cancer has been confirmed, but further verification in animal model experiments is still required (Butt et al., 2021).

### 3.7 Therapeutic effects of taxifolin in endocrine system

#### 3.7.1 Therapeutic effects of taxifolin in regulate glucose metabolism

**FIGURE 8:**
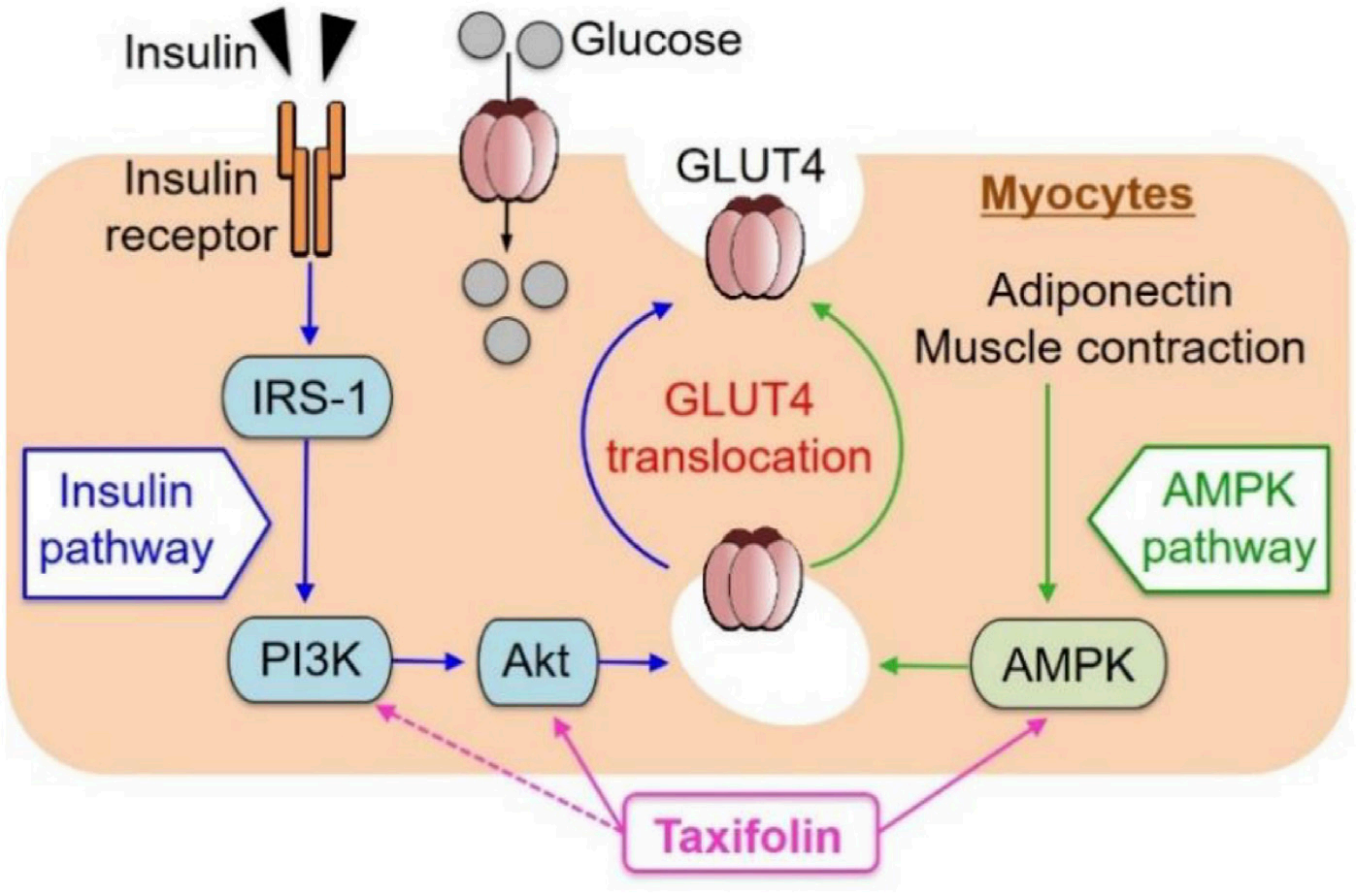
Schematic representation for possible factors involved in glucose uptake by myocytes from extracellular glucose pools (Kondo et al., 2021). Reproduced with permission from Kondo et al., 2021. A dose-dependent increase in GU was observed in cultured L6 myotubes following the application of taxifolin through the PI3K/Akt and AMPK signaling pathways. Translocation of GLUT4 to the plasma membrane was promoted by activation of each pathway independently.

Taxifolin has been shown to be effective in treating diabetes. In recent years, new advances have been made in the regulation of glucose metabolism and the prevention and treatment of diabetes complications. Skeletal muscle is responsible for the majority (about 75%) of postprandial insulin-mediated glucose uptake and therefore plays an imperative role in glucose balance (Saltiel and Kahn, 2001). Kondo et al. investigated the effect of taxifolin on glucose metabolism in L6 muscle cells (myotubes). It has been demonstrated that taxifolin activates Akt and AMPK and facilitates the transport of glucose transporter 4 (GLUT4) from the cell membrane of the L6 myotube to the plasma membrane. Taxifolin dose-dependently increases glucose uptake in L6 myotubes. *In vivo* experiments have also confirmed that taxifolin can significantly improve fasting plasma glucose, insulin, uric acid levels and an index of insulin resistance (HOMA-IR) of KK-Ay/Ta mice with hyperglycemia and hyperuricemia (Kondo et al., 2021) (Figure 8).

Taxifolin inhibits α-glucuronidase, α-amylase and pancreatic lipase. Pre-administered taxifolin can significantly reduce postprandial hyperglycemia in rats in vivo studies. In addition, it can also reduce the absorption of triglycerides by inhibiting pancreatic lipase (Su et al., 2020).

Taxifolin is not only effective in improving glucose metabolism, but also in improving complications associated with diabetes. The condition known as diabetic retinopathy (DR) is one of the leading causes of blindness in people with diabetes mellitus, and taxifolin may be beneficial in treating diabetic retinopathy. As a result of treating rats with a blood glucose level of 250 mg/dL with taxifolin, Ahiskali et al. studied the protective effect of taxifolin on diabetic retinopathy from a biochemical and histopathological perspective. Blood samples showed partial recovery in levels of Malondialdehyde (MDA), tGSH, IL-1β and TNF-α. In the taxifolin group, the retinal ganglion cells are slightly dilated, and the blood vessels are congested; In the diabetes control group, the retinal ganglion cells are severely damaged (Ahiskali et al., 2019). Additionally, taxifolin can relieve urea-induced hyperglycemia-related neuropathy and neuropathic pain. According to Alay et al., albino Wistar male rats were injected intraperitoneally with 120 mg/kg of alloxan, causing hyperglycemia, followed by treatment with taxifolin. Based on the results of biochemical and histopathological examinations, taxifolin significantly reduced the increase in blood glucose and the decrease in the threshold of paw pain. There was also a significant reduction in the oxidant-antioxidant balance in sciatic nerve tissue, as well as a deterioration in the morphology of animal tissues (Alay et al., 2022).

#### 3.7.2 Therapeutic effects of taxifolin in regulate glucose metabolism

The balance of bones is maintained by the interaction of osteoclast bone resorption and osteoblastic osteogenesis, and due to its anti-inflammatory properties, taxifolin has significant advantages in the regulation and balance of bone metabolism. Taxifolin has been shown to stimulate osteoblastic differentiation of bone marrow mesenchymal stem cells by inhibiting nuclear translocation of NF-κB in previous studies (Wang et al., 2017); Taxifolin can also promote osteoblastic differentiation of MC3T3-E1 cells; and inhibits osteoclastogenesis in RAW264.7 cells (Satué et al., 2013).

According to Cai et al., taxifolin prevents bone loss in osteoporosis mice and clarifies the specific mechanism by which taxifolin suppresses osteoclastogenesis. This study examined the effects of taxifolin on osteoclast production and function both *in vitro* and *in vivo*. It was found that taxifolin inhibited osteoclastogenesis by modulating several nuclear factor-κB ligand (RANKL) signaling pathways; in addition, taxifolin decreased the expression of osteoclast-specific genes, including Trap, Mmp-9, Cathepsin K, C-Fos, Nfatc1, and Rank. Moreover, taxifolin alleviated bone loss caused by oophorectomy by suppressing activity and reducing *in vivo* serum levels of tumor necrosis factor-α, interleukin-1β, interleukin-6, and receptor activator of RANKL (Cai et al., 2018). In another study, *in vitro*, taxifolin inhibited RANKL-induced osteoclast differentiation in human bone marrow-derived macrophages (BMMs) without cytotoxicity; *In vivo*, taxifolin also prevented bone loss as assessed in a mouse calvarial osteolysis model (Zhang et al., 2019b).

Lektemur Alpan et al. investigated the effects of taxifolin on ligation-induced experimental periodontitis in rats and ligation-induced experimental periodontitis in diabetic rats, respectively. Based on the results of the two experimental CBCTs, taxifolin inhibited alveolar bone loss. In addition, taxifolin increased the expression of BMP-2, OCN, ALP, and Col1, while decreasing the expression of RANKL. Furthermore, in the taxifolin group, the expression of Bcl-2 increased, while that of Bax decreased. It can be seen that taxifolin has a therapeutic effect on both periodontitis caused by rat ligation and periodontitis in diabetic rats (Lektemur Alpan et al., 2020; Lektemur Alpan et al., 2022).

### 3.8 Therapeutic effects of taxifolin in reproductive system

On the one hand, Bedir et al. investigated the effects of taxifolin on experimental testicular ischaemia reperfusion injury in rats and performed biochemical and histopathological analysis. Based on the biochemical results, taxifolin inhibited the increase in MDA content and the decrease in tGSH and superoxide dismutase (SOD) levels in the testicular caused by ischaemia reperfusion injury. Histopathological results, however, demonstrated that taxifolin alleviated damage caused by ischemia reperfusion injury to germinal epithelium cells and seminiferous tubule (Bedir et al., 2022). In addition, Li et al. demonstrated that taxifolin was also effective in attenuating the developmental testicular toxicity induced by di-n-butyl phthalate in fetal male rats (Li et al., 2020).

On the other hand, taxifolin is beneficial in the treatment of oxidative ovarian damage, infertility, as well as reproductive dysfunctions induced by clozapine (CLN) and haloperidol (HPL). Ince et al. examined the effects of taxifolin on oxidative ovarian damage and reproductive dysfunction in female rats induced by antipsychotic drugs. It was demonstrated that taxifolin significantly reduced the growth of MDA and tGSH levels, alleviating histopathological damage (Ince et al., 2021).

### 3.9 Therapeutic effects of taxifolin in urinary system

Taxifolin exhibits antifibrotic activity, but its pharmacological mechanism is unclear. According to Ren et al., who studied unilateral ureteral obstruction (UUO) and its possible mechanisms, serum metabolic analysis using UPLC-Q-TOF/MS showed that 32 potential biomarkers were associated with renal fibrosis (RF), 27 of which were regulated by taxifolin regulation (Ren et al., 2020). Similarly, to investigate the mechanism of taxifolin anti-fibrosis, Wang et al. performed *in vivo* and *in vitro* studies, choosing unilateral ureteral obstruction (UUO) mice for the *in vivo* model and NRK-49F cells for the *in vitro* study. Unlike Ren et al., Wang et al. focused their study on Changes in fibroblast activation, collagen synthesis, oxidative stress, and related signaling pathways by immunohistochemical staining, Western blot analysis, real-time reverse transcription-PCR, and fluorescence microscopy. Ultimately, it was similarly verified that taxifolin had significant preventive and therapeutic effects on UUO-induced renal fibrosis and inhibited fibroblast activation by reducing oxidative stress and Smad3 phosphorylation *via* Nrf2 signaling (Wang et al., 2020). Changes in fibroblast activation, collagen synthesis, oxidative stress, and related signaling pathways.

Colistin is an antimicrobial agent used to treat resistant Gram-negative infections. Its primary adverse effect is nephrotoxicity, and the combination of taxifolin and dapagliflozin may reduce Colistin-induced nephrotoxicity (Kabel and Salama, 2021). In response to acrylamide-induced nephrotoxicity, taxifolin can inhibit the increase of MDA, IL-1β and TNF-α, and reduce the level of tGSH associated with acrylamide, effectively preventing and treating acrylamide-induced kidney damage (Bedir et al., 2021). Additionally, taxifolin can inhibit overactivity of the renin-angiotensin-aldosterone system (RAAS) and improve glucose metabolism and water-salt metabolism in rats with metabolic syndrome through its activity on the PI3K/AKT signaling pathway (Gao et al., 2020).

## 4 Outlook

As explained above, taxifolin has various pharmacological activities and significant therapeutic potential for the human body. In spite of this, most trials have been conducted at the cellular and animal level, revealing only the benefits of preclinical studies, while clinical trials have not been conducted so far. At the same time, it still has some deficiencies, such as poor stability, permeability, and low bioavailability, which limit its use. *In vitro* forced degradation tests and *in silico* stability predictions showed that taxifolin was extremely unstable under alkaline hydrolysis, and that the alkaline degradation product was a dimer of taxifolin (Stenger Moura et al., 2021b). There have been other studies which suggest that taxifolin is limited in its ability to cross the blood-brain barrier (BBB), limiting its effectiveness against amyloid β aggregation in the brain (Yang et al., 2016). Drug absorption is primarily limited by the single layer of intestinal epithelial cells that cover the luminal surface of the intestinal wall. The majority of drugs are absorbed *via* passive transcellular transport. However, taxifolin is a poorly soluble natural compound, resulting in poor bioavailability (Artursson and Karlsson, 1991; Shikov et al., 2009). Studies have shown that oral taxifolin has a wide range of bioavailability in rats. A dose of 10–100 mg/kg body weight of taxifolin administered orally resulted in very low plasma concentrations in rats, and the bioavailability was measured at 0.17% in comparison to intravenous administration. In another study, A et al. administered a single oral dose of taxifolin at 12.5, 25 or 50 mg/kg or injected 50 mg/kg intravenously with taxifolin. They found that the bioavailability of oral taxifolin was 24% compared to intravenous injection. Studies on the bioavailability of drugs in humans are limited, with *in vitro* studies using human skin samples showing a bioavailability of 45 percent (Alves et al., 2018). These may be reasons for neglect or failure in further clinical trials. Therefore an appropriate drug delivery system should be considered when applying taxifolin in order to maintain its stability, promote its permeability, and enhance its bioavailability.

In addition, there is evidence that the effective form of taxifolin is not only the parent form, but also its metabolites produced *in vivo*, which can exert their *in vivo* effects simultaneously or sequentially with taxifolin (Cai et al., 2015). To investigate its metabolism *in vivo*, an HPLC-ESI-IT-TOF-MS(n) method combined with specific metabolite detection strategy was used to detect and identify taxifolin metabolites in rats. Among them, the activities of 17 bioactive metabolites can cover almost all biological activities of taxifolin, and even some metabolites have the same target as taxifolin (Yang et al., 2016). This may explain that taxifolin still works in animals despite its low bioavailability. Therefore, for further clinical applications, further studies on taxifolin metabolites and biotransformation in humans are needed.

Besides, flavonoids exhibit pleiotropic effects rather than targeting individual targets (Pohl and Kong Thoo Lin, 2018). Different targets may have opposite effects or may act synergistically through different signaling pathways. When investigating the anticancer activity of taxifolin, taxifolin treatment resulted in a dose-dependent increase in ZEB2 protein expression. However, taxifolin treatment also reduced ZEB2 signaling by inhibiting Akt phosphorylation (Zdenek et al., 2021). A multi-targeted, non-specific effect of Taxifolin may result in ambiguous or even contradictory biological effects. To develop a drug for human use, it is recommended that further research be conducted on the molecular mechanisms involved and the safety profile using well-designed randomized trials.

In view of the above, future research should focus on the following elements related to taxifolin:

Firstly, due to the poor stability, permeability, and low bioavailability of taxifolin, its application should consider loading an appropriate drug delivery system and focusing on its metabolites and biotransformation in humans; Secondly, due to the pleiotropic nature of taxifolin, more research needs to be conducted on its pharmacokinetic profiles, profound molecular mechanisms, and drug safety standards in the context of well-designed randomized clinical trials.

Lastly, it should be noted that more attention should be paid to its clinical application research in the future, given its antioxidant, anti-inflammatory, antibacterial, and other properties. For instance, taxifolin may be used in dentistry to preserve extraction sockets, promote osseointegration of implants, assist GBR treatment, and to serve as an antibacterial mouthwash.

As a whole, taxifolin has positive pharmacological activities and a favorable therapeutic effect. It is crucial to continue well-designed research and clinical application to meet the daily-life and treatment needs of the human body.
